# The Genetic Code Assembles via Division and Fusion, Basic Cellular Events

**DOI:** 10.3390/life13102069

**Published:** 2023-10-17

**Authors:** Michael Yarus

**Affiliations:** Department of Molecular, Cellular and Developmental Biology, University of Colorado, Boulder, CO 80309-0347, USA; yarus@colorado.edu

**Keywords:** RNA world, SGC, evolution, code accuracy, Monte Carlo kinetics

## Abstract

Standard Genetic Code (SGC) evolution is quantitatively modeled in up to 2000 independent coding ‘environments’. Environments host multiple codes that may fuse or divide, with division yielding identical descendants. Code division may be selected—sophisticated gene products could be required for an orderly separation that preserves the coding. Several unforeseen results emerge: more rapid evolution requires unselective code division rather than its selective form. Combining selective and unselective code division, with/without code fusion, with/without independent environmental coding tables, and with/without wobble defines 2^5^ = 32 possible pathways for SGC evolution. These 32 possible histories are compared, specifically, for evolutionary speed and code accuracy. Pathways differ greatly, for example, by ≈300-fold in time to evolve SGC-like codes. Eight of thirty-two pathways employing code division evolve quickly. Four of these eight that combine fusion and division also unite speed and accuracy. The two most precise, swiftest paths; thus the most likely routes to the SGC are similar, differing only in fusion with independent environmental codes. Code division instead of fusion with unrelated codes implies that exterior codes can be dispensable. Instead, a single ancestral code that divides and fuses can initiate fully encoded peptide biosynthesis. Division and fusion create a ‘crescendo of competent coding’, facilitating the search for the SGC and also assisting the advent of otherwise uniformly disfavored wobble coding. Code fusion can unite multiple codon assignment mechanisms. However, via code division and fusion, an SGC can emerge from a single primary origin via familiar cellular events.

## 1. Introduction

**The problem.** Automated total searches of ≈2.5 × 10^5^ bacterial and archaeal genomes find [1,2] only slightly altered genetic codes, related to the Standard Genetic Code (SGC). Hence, true alternative codes are exceedingly rare on modern Earth. Modern biota, therefore, are convincingly traced to a single ancestral group encoding peptides using a close SGC relative. This ancient, all-inclusive ancestor presents a problem of ultimate significance for biology and is the topic here.

**Early coding.** The code’s origin presents an inevitable succession problem: one seemingly cannot evolve proteins, like aminoacyl-RNA synthetases (AARS) without prior competent coding/protein synthesis. AARS are complex amino acid polymers binding several substrates, performing stereo- and regiospecific chemistry. Thus, one must evolve complex, accurate peptide synthesis using precursors of modern protein AARS, presumably precursors composed of RNA. Thus, RNA world evolution for AARS is implied. This work characterizes this early period before the appearance of the protein AARS and their later complex enzymatic evolution. 

**Incorporating code division.** We follow the evolution of numerous RNA-based coding tables through time in code evolution environments, using Monte Carlo kinetics [3], see Section 4. The time for one round of evolution in a code environment is called a passage. An evolving environment undergoing a passage containing zero, one, tens, or hundreds of coding tables can either initiate coding (probability = Pinit) in the first table with a random assignment, add a new table (Ptab), begin by assigning a random codon to a randomly chosen function (20 amino acids, start, stop), or the environmental code can divide (Pdiv), adding an additional code identical to the pre-existing one. Code division can be non-selective, allowing any code to divide, or a completeness criterion (cc) can specify that division occurs only after a certain variety of encoded functions that are possible. A completeness criterion recognizes that specific folding [4] and enzymatic function [5,6,7] are observed only after polypeptides contain 10–13 different amino acids. A proteome capable of precise division, therefore, plausibly requires prior encoding of that number of amino acids.

A world may comprise many contemporaneous environments and report its status when an environment reaches a set goal, such as SGC-level completeness (e.g., encoding ≥20 functions) or at a specified time (e.g., after 121 passages).

## 2. Methods

**Monte Carlo kinetics.** Imagine recording events by quickly making a mark on a chart moving past you across a table: for coding evolution, these marks represent codon assignments, related codon captures, code divisions, and so on. The table is not passive: it both records marks and allows them to evolve according to physical and chemical laws. At the end of evolution, lay a laddered grid over your series of chart marks. The ladder has narrow gaps; only one event (or no event) ever appears in an inter-step opening of your ladder. In placing your grid, you have not changed the sequence of events and, notably, not changed the evolutionary result on the table. Thus, you have shown that you can reproduce a “continuous” kinetic process with small adjacent windows (here called passages), assigning probabilities for each kind of event within a window. If the probability of a mark on the chart depends on the concentration of something on the table, you are modeling first-order kinetics and defining a first-order rate constant [3]. If the probability of appearing in a window/passage depends on two concentrations, you are modeling second-order kinetics. This Monte Carlo framework makes it simple to implement many rates you wish to study, even in complex environments like that in a (coding) table, with many changes occurring at many loci for change. The resulting source code in Pascal is available on request from the author. An array of 32 named pathways with selected results is in a Supplementary Data File, Table S1.

**Tracking time.** Time passes for environmental codes in passages. During a passage, every existing coding table is given a chance to either assign a randomly chosen, unassigned codon (probability Pinit), have a previous assignment decay (Pdecay), or capture an unassigned codon a single mutation distant (Pmut), conferring on it an assignment with a closely related polar requirement [8,9], including transferring the assignment of the initial codon. The final option is that before internal passages, two coding tables may fuse (Pfus)—this can augment code growth if the fusees combine compatible assignments, or it can yield extinction of fusees if their pre-existing codes make differing assignments to the same codon. Such incompatible, ambiguous codes are inactivated and do not evolve further [10]. Alternatively, an existing code accurately divides (Pdiv) during a passage. 

**Coding and wobble coding.** Functions are encoded by assignment to a single coding triplet or via simplified Crick wobble, which allows U/G and G/U pairs at the third position of codons [3,11]. The onset of wobble is controlled by Pwob, the probability of wobble onset per passage [12]. 

## 3. Results

**Figure 1:**
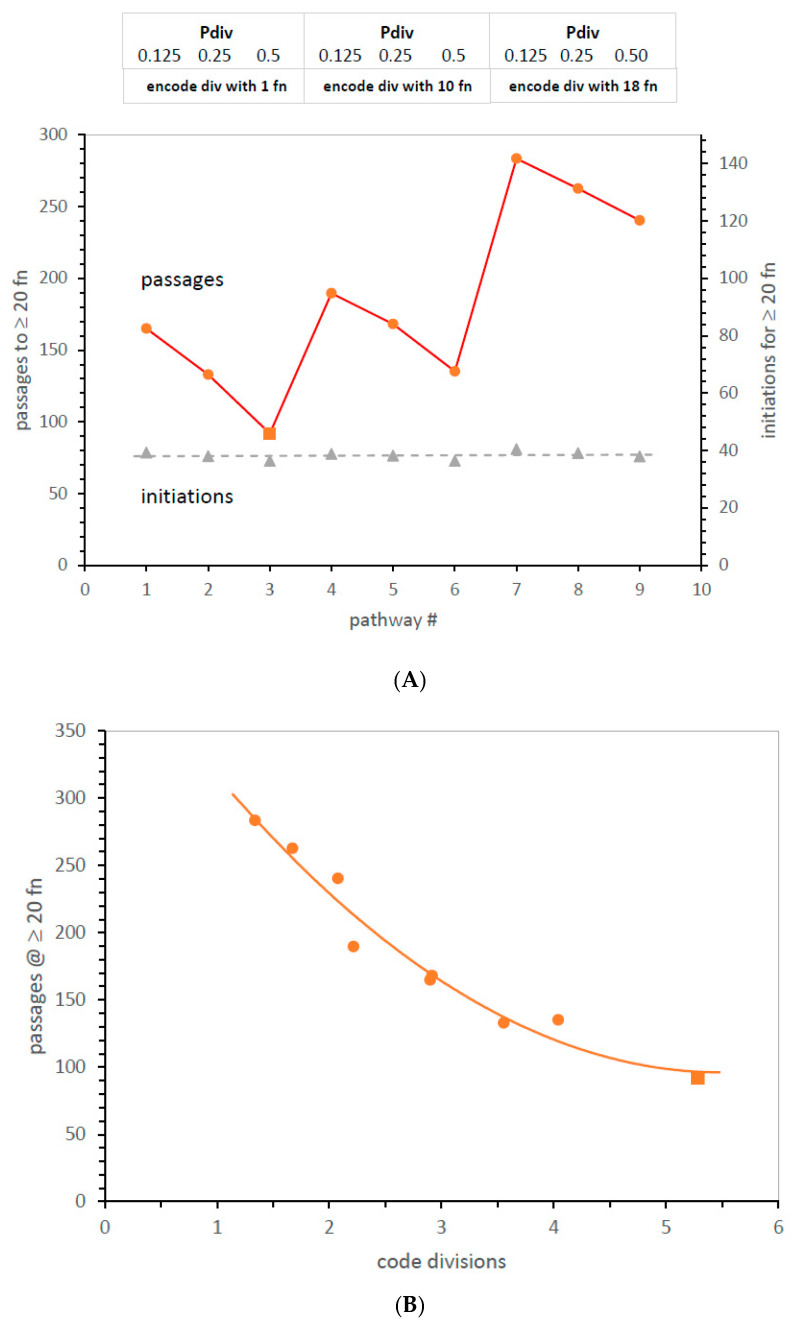
(**A**). Effects of code division on time to evolve ≥20 encoded functions, and on number of initial assignments required for ≥20 encoded functions. The pathway x-axis follows a structured list of code division variables (see text) named in the box at graph top: “Pdiv” = probability of unselected code division/passage, “encode div with” = code completeness (cc) required to encode accurate code division. Pmut = 0.00975, Pdec = 0.00975, Pinit = 0.15, Prand = 0.05, Pfus = 0.001, Ptab = 0.08, Pwob = 0.0—results show means for evolution in 500 environments. (**B**). Mean time to evolve ≥20 encoded functions versus mean number of code divisions for those codes. A square marks shortest evolutionary time. Environments are those in (**A**).

**Presenting effects of code evolution.** Worlds composed of perhaps hundreds of environments with variably evolving codes present problems of exposition. Multiple presentation problems can be solved by the method in Figure 1A. Mean data are listed in a specific numbered order (#1, 2, 3, etc.) reflecting groupings of underlying mechanisms; thus, the effects of the mechanism can be read from periodicity in the plot. Multiple mechanistic effects are evident in an ordinary two-dimensional figure.

Structured plotting is illustrated (Figure 1A) by three groups of codes that have a different threshold for code division (cc, the completeness criterion) at 1, 10, and 18 codon assignments. Within each such group, codes have an unselective probability of division per passage of 0.125, 0.25, and 0.5. Thus, one can read the effects of increasing division within groups of three and also read the effects of different division thresholds by comparing such triple groups. 

**Effects of code division.** In Figure 1A, increased division (Pdiv) always reduces the time to evolve SGC-like coding; that is, ≥20 assigned functions. Quicker evolution is slightly less for the same Pdiv change at a higher threshold (comparing mean slopes of threes). In addition, evolution is increasingly rapid if the division threshold is lowered from near-completion (set at ≥20 functions encoded) to no threshold at all on the left (threshold at one function; any code can divide). Figure 1A, therefore, presents a non-trivial result: non-selective code division (mechanism #3, red square), acting throughout evolution, evolves SGC-like coding the fastest. 

**Division and rate of evolution.** Division is revisited in Figure 1B, plotting the speed of evolution versus the number of code divisions to reach an SGC-like assignment. In Figure 1B, the time to ≥20 encoded functions with Figure 1A’s variety of division probabilities and thresholds declines rapidly as code division increases. The fastest mean SGC-like evolution with code division, 92 passages, is more than three times faster than previous environments with similar code passage probabilities [10,13]. 

**Small effects.** A mechanism-structured plot is also useful when substantial effects are absent. Figure 1A plots the number (≈38) of random initial codon assignments required to reach ≥20 different assigned functions on its rightward axis. This is hardly altered in mechanisms one through nine. Close inspection of displacements from the least-squares dashed line discloses periodic behavior; fast evolution requires slightly fewer assignments. However, the structured plot (Figure 1A) highlights how small this effect is for these changes in code division. Fixed assignments are not a rule; pathways can use assignments inefficiently. Still, even conditional assignment constancy will be useful below in clarifying complex evolution. 

**Presenting code accuracy.** A general measure of SGC similarity is frequently useful. One would like to avoid assigning an SGC-like number of codons but to different functions than in the actual SGC.

In this work, misassignments (abbreviated “mis”) with respect to the biological SGC are counted. Codes with no difference from SGC assignment are denoted mis0, those with one difference are mis1 codes, and on to mis2, mis3… The fraction of SGC-like assignments provides an index of distance that meets our need to measure evolutionary accuracy.

**Figure 2:**
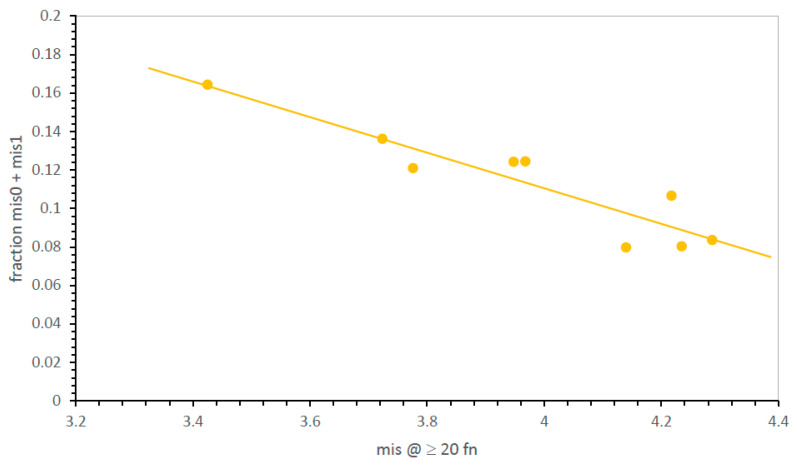
Mean mis and mean fraction of codes with SGC-like assignments are closely, and inversely, related. mis0 = identical to SGC assignments; mis1 = one difference from SGC assignments. Environments are those in Figure 1A.

However, this pose a problem of precision: SGC-identical, mis0 codes can be infrequent, even pragmatically unmeasurable for inaccurate evolutionary modes. However, Figure 2 shows how this problem can be met. The distribution of errors is smooth and unimodal—the fraction of SGC-like codes (here, the fraction that is mis0 and mis1) rises smoothly with the decrease in mean mis in near-complete codes. Because mean mis are measured in up to two thousand environments, average misassignment is usually known with precision. Proximity to the SGC is therefore measured (Figure 2) either by calculating mean misassignment (mis; accuracy better when smaller) among most complete codes or by counting codes nearest the SGC when accessible (mis0, mis1—accuracy better when larger). 

**Figure 3:**
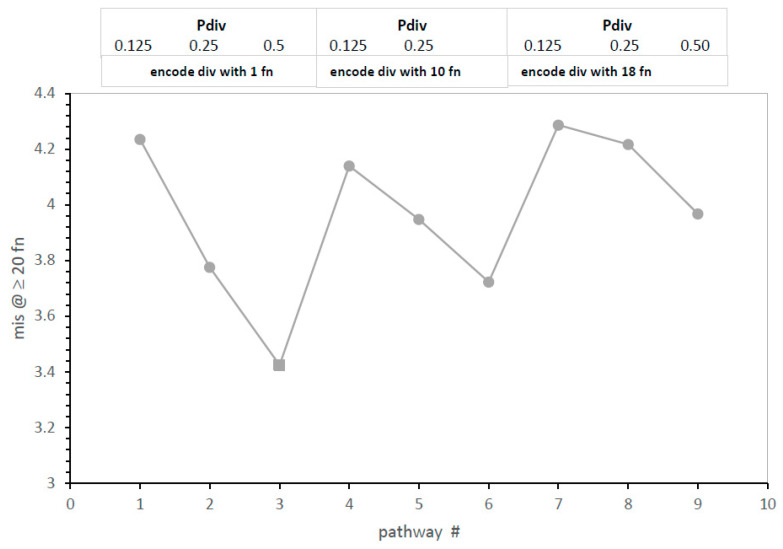
Effects of division variables (Pdiv and cc) on accuracy of evolution to ≥20 encoded functions. The x-axis is a structured list of pathways like in Figure 1A. Environments are those in Figure 1A. A square marks the most accurate pathway.

**Mechanisms and code accuracy.** Accuracy as mean misassignments in Figure 3, like time in Figure 1A, is plotted versus Figure 1A’s division pathways one to nine. A Figure 1A-like pattern reappears. Therefore, code accuracy is greatest with more division (greater Pdiv) in several contexts. The sensitivity of code accuracy to division frequency declines significantly as a division threshold increases (Figure 3). Absolute accuracy is also greatest when the threshold (completeness criterion, cc, square marker) is low: pathway #3 is the most accurate (Figure 3). Most accurate code evolution utilizes frequent division and approaches the SGC quickly without selection for coding sophistication; any code at all meets a one-assignment division “threshold”. This result reappears in a much more complex mechanistic context below.

Moreover, in Figure 3, code division has an interesting property previously shown for code fusion [10]: more division (greater Pdiv) reduces error, implying constraint of the present mixture of initial SGC and random assignments. Such adherence to an underlying coding consensus (Figure 3) is weakened if a threshold delays the initiation of code division. However, more code division, not division selecting code progress, produces an accurate code (Figure 3) while also evolving it quickly (Figure 1A).

**Figure 4:**
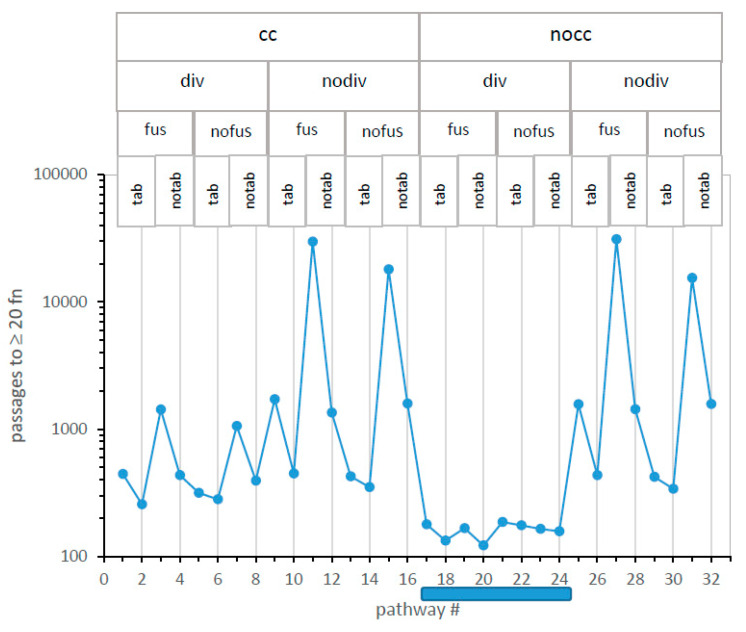
Logarithmic mean time to evolve ≥20 encoded functions for 32 potential code evolution pathways. Pathway mechanism abbreviations are listed at graph top:.cc = require completeness criterion for code division, nocc = no cc required; div = allow code division with probability Pdiv, nodiv = no code division; fus = allow codes to fuse with probability Pfus, nofus = no code fusion; tab = allow independent environmental coding tables, origin probability Ptab; notab, no parallel tables; wob = allow wobble coding (no vertical line through point), nowob = simple base pairing (vertical line); A shaded bar below the x-axis marks the favored nocc div canyon. Numerical data are presented in a Supplementary Data File, RFW_supp_data_a.xlsx.

**Five-dimensional comparison of 32 pathways.** Incorporation of division effects into a Monte Carlo kinetic scheme (Methods) for specific code table evolution defines 32 pathways toward the genetic code: with/without code division (probability of division, as well as division threshold), with/without code fusion [10], with/without independent coding tables [10], and with/without simplified Crick wobble [3]. The 32 pathways are quantitatively compared in Figure 4, using the structured display method of Figure 1A to organize five-dimensional data (see the Supplementary File). 

Figure 4 presents the time to reach ≥20 encoded functions (in passages, ordinate) versus all 32 numbered mechanisms on the x-axis. For example, minimal time to evolve ≥20 encoded functions occur via mechanism #20, which (reading titles above and the vertical line through the point: legend, Figure 4) utilizes no completeness threshold for division (nocc), incorporates probable code division (div), allows codes to fuse (fus) but has no independent codes forming in its environment (notab), and evolves during initial assignments in the absence of wobble (nowob vertical line). Path #20 reappears frequently below.

**A glance identifies the fastest evolution.** In Figure 4, mechanisms that have no completeness threshold (nocc: cc = 1) and probable code division (div) form a “canyon” (mech #17–24; shaded bar), each of whose eight pathways evolve ≥20 encoded functions faster than any of the other 24 paths examined. 

Moreover, this nocc div canyon is the major difference between Figure 4 left and right. Superior unselective code division, first seen in Figure 1A, reappears here in a broader mechanistic context. Therefore, the path of least selection [14], that is, the probable evolutionary path, will be a nocc div unselective route. Accordingly, code division greatly changes early code evolution, and nocc and div will be necessary elements in the best SGC pathways.

**A glance identifies the slowest evolution.** In Figure 4, the four slowest routes to the SGC have in common that codes do not divide (nodiv), and no additional codes appear alongside independent code origins (notab). Under such conditions, fusion is irrelevant because there are no additional codes to fuse. Thus, for these four slowest pathways, fus/nofus mechanisms are about equivalently poor because code fusion is inaccessible and irrelevant. A single code in each environment must evolve alone to SGC proximity, and this requires a complex set of events, with many digressions, making these the most improbable evolutionary routes. This matches previous observations [10] and rationalizes the superior pathways considered below, all of which exploit code-code interactions.

**Wobble is always inhibitory.** Among 32 pathways in Figure 4, 16 encode using wobble, and 16 do not. One can consider the 16 wob (no vertical line)/nowob (line) pairs together by noting that each wobbling pathway (no vertical line) is accompanied by a non-wobbling pathway immediately to its right (line) that differs only in lacking a simple Crick wobble [3]. 

Mechanisms differ in their sensitivity to wobble. Slow single-coding-table environments are very much impaired if they must use wobble assignments. In contrast, the eight mechanisms of the nocc div canyon (Figure 4) are less sensitive to inhibitory wobble effects. However, throughout all 16 wob/nowob pairs in Figure 4, wobble prolongs evolution to the SGC in 16 varied mechanistic contexts. This extends previous findings that assignments that commit more triplets always impede progress toward complete coding [13,15] and that wobble specifically disrupts the evolution of codes that most resemble the SGC [12]. Figure 4′s kinetics strongly reinforce previous structural arguments; accurate wobble requires a complex ribosomal isomerization [10,16] and a complex functional tRNA structure [17,18]—thus wobble encoding probably appeared late in RNA code evolution, after most functions.

The addition of simple Crick wobble to present codes adds, minimally, two misassignments because unique SGC encodings, AUG/Met and UGG/Trp, are not accounted for here. Unique assignments are most simply explained as survivors from the early non-wobbling era defined just above. However, an essential code transition from unique to wobbling assignments can definitely bear more thought.

**Independently originating codes (tab) speed SGC evolution, but not in the nocc div canyon.** The effect of multiple independent codes arising side-by-side, then interacting within an SGC-evolving environment, can also be assessed in Figure 4. Pairs of tab/notab mechanisms, in which the only change is the absence of independently evolving coding tables, have sequential odd or even numbers. 

For example, mechanisms #10 ⇔ #12 and #18 ⇔ #20 differ only in lacking parallel environmental codes in the higher-numbered mechanisms. However, the two code pairs differ greatly in the resulting effect. Loss of other codes slows SGC evolution significantly on the left in Figure 4 (#10 to 12; 447 to 1345 passages), where nodiv cuts off other codes arising by division. In contrast, on the right (#18 to 20; 133 to 121 passages), with a supply of alternative fusion partners available from code division, parallel independent codes are instead slightly inhibitory to evolutionary progress. Similarly, for all codes in 12 tab/notab pairs outside the canyon and each of 4 such pairs within the #17 to 24 canyon, codes arising by code division are always more favorable partners than independent coding tables. This gathering of coding information from several into one nascent code returns in the discussion section.

**Figure 5:**
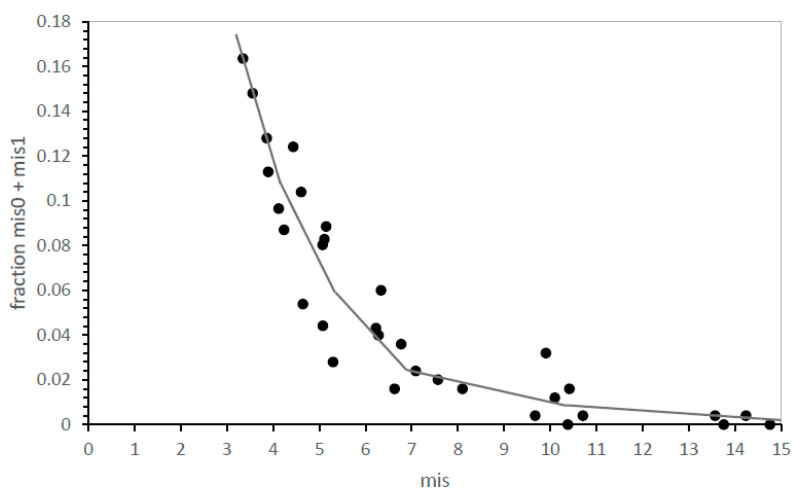
SGC-like encoding (mis0 and mis1) versus mean mis at ≥20 encoded functions. Environments are those in Figure 4.

**Speed and accuracy are related.** Given that genetic codes can adhere to underlying consensus assignments [10], the existence of such adherence (as in Figure 3), as well as evolutionary speed (as in Figure 4), is of importance. For the highly varied 32 possible mechanisms, as for the smaller, more uniform group of code divisions (Figure 2), the fraction of codes near the SGC increases as the mean number of misassignments declines. That is, the distribution of error regularly sharpens as the mean misassignment in ≥20 function codes declines, drawing in toward an SGC consensus. In Figure 5, paralleling Figure 2 for division variation only, mean misassignment (mis) is a useful measure of SGC proximity, represented as the sum of mis0 and mis1 code fractions. In fact, SGC-like codes increase more rapidly as mean misassignment closes in on the SGC, yielding a very sensitive index of SGC proximity (Figure 5).

**Figure 6:**
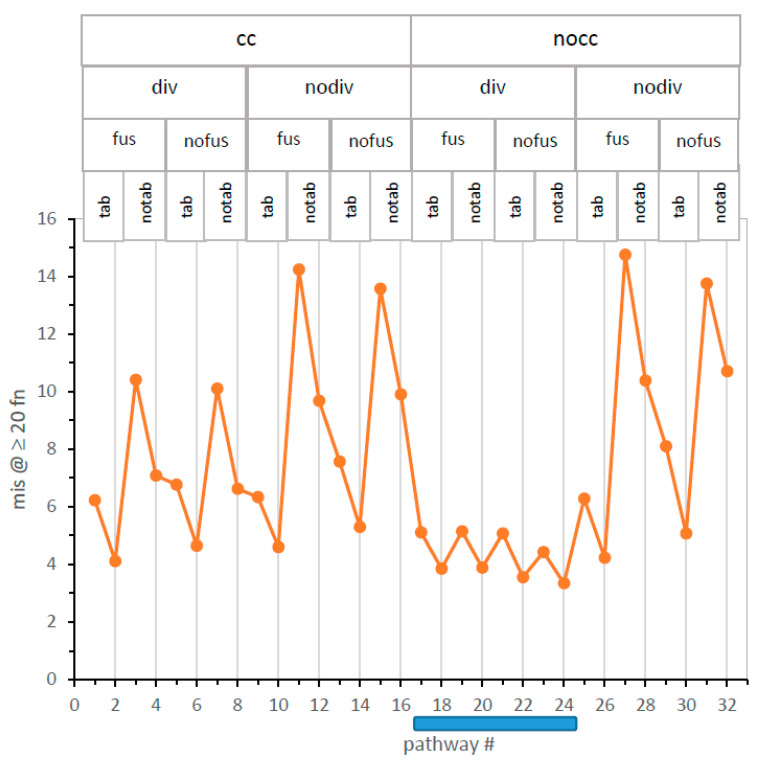
Mean misassignment (mis) at ≥20 encoded functions for 32 code pathways. Pathway mechanism abbreviations are listed at graph top. Environments are those in Figure 4. The shaded bar beneath the x-axis marks the favored nocc div canyon.

In Figure 6, mis is plotted for the complete structured set of 32 pathways. A comparison of mechanism-structured plots in Figure 4 and Figure 6 shows that evolutionary speed and accuracy are related; the two plots are similar over most pathways. For example, there is again a mechanism #17–24 nocc-div canyon, within which the lowest global code error appears. However, small differences in speed and accuracy from independent tables are observed (e.g., pathway #9). 

**Figure 7:**
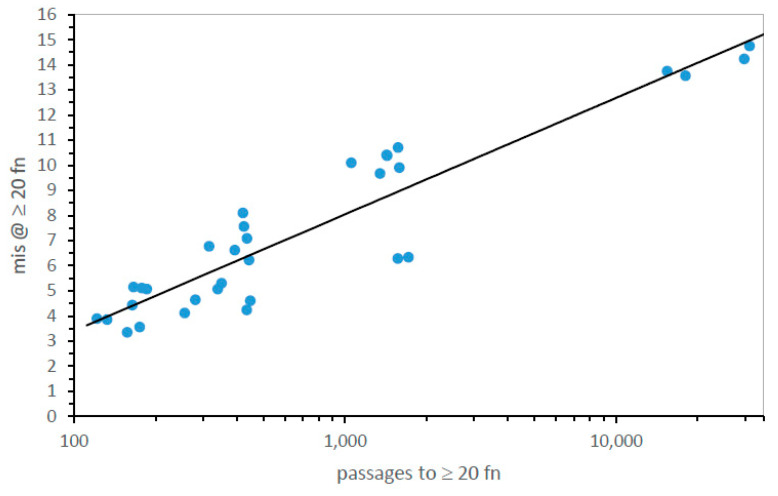
Mean misassignment at ≥20 encoded functions versus time for its evolution in passages, for 32 code evolution pathways. Environments are those in Figure 4.

Figure 7 makes explicit this interaction between speed and accuracy by plotting time to evolve ≥20 encoded functions in passages vs. resulting mis. There is a clear relation, though with some variation: the least squares line accounts for 86% of the variance in misassignment. Therefore, fast evolution tends to occur using pathways that also approach SGC consensus. Figure 1A, Figure 3, Figure 4, and Figure 6 convey a decisive property of code evolution: it is not necessary to choose between rapid code evolution and code adherence. There are quick routes to codes that are also SGC-like. 

More quantitatively, mechanisms #18 and 20 most quickly present near–complete codes (Figure 4). These pathways have low levels of misassignment: more than a quarter of all ≥20 function codes are 0, 1, or 2 assignments from the SGC. In fact, codes identical to the SGC (mis0) are more than 1 in 40 of these near-complete coding tables. Nocc-div canyon codes again provide the least selection, that is, an evolutionary route favored because it requires the least selected alteration to become the SGC [14].

**Distinguishing canyon codes.** To focus discussion, mechanisms #18 and 20 are put foremost because they most rapidly produce complete coding (Figure 4). As Figure 6 shows, they do not precisely correspond to maximal resemblance to the SGC; canyon pathways 22 and 24 have slightly greater mean SGC similarity.

Differences between canyon codes appear small but are significant. Between ≥20 functions in mechanisms #20 and #18, 11.2 passages intervene. Given their standard errors in 1000 environments each, a two-tailed, unequal variance t-test yields 1.8 × 10^−15^ as the probability that these mean times are the same. Thus, the time profiles in Figure 4 convey statistically valid differences. Pathway #20 really arrives at ≥20 encoded functions before #18. However, this significance leaves open an essential question. 

**Figure 8:**
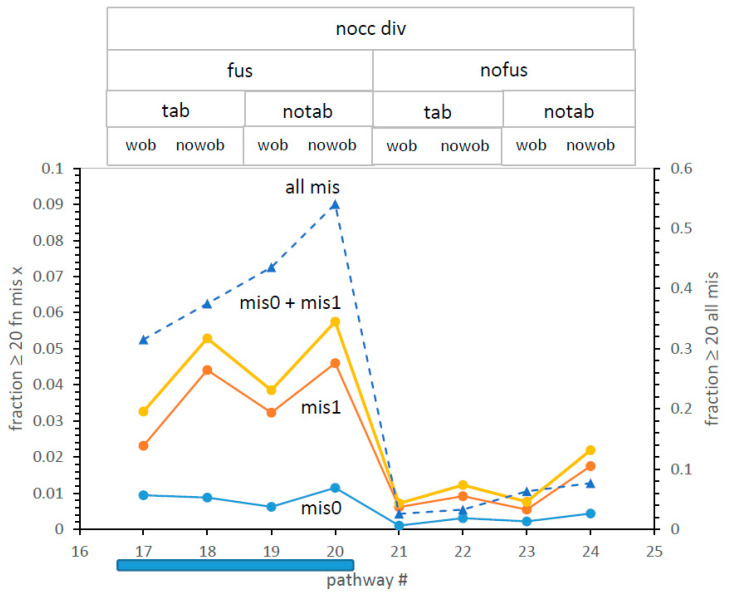
Fraction of codes with both ≥20 encoded functions and mis0 or mis1, or ≥20 encoded functions with any mis. All environments have run for 121 passages, the mean time for pathway #20 to reach ≥20 encoded functions. The shaded bar beneath the x-axis marks the superior nocc div fus section of the nocc div canyon pathways. Conditions are those of Figure 1, except Ptab = 0.08 or 0.0, Pwob = 0.005 or 0.0. Fractions are mean proportions of 1000 environments.

**What code differences are significant?** Are Figure 4′s time differences, however statistically significant, of importance to evolution? This question can be approached quantitatively using the notion of least selection [14]. Figure 8 combines code completeness and accuracy in one metric. The abundance of codes that both encode ≥20 functions (completeness) and are accurate (fewest differences from SGC assignments) is taken as the distance to be crossed by selection. This is most relevant at early times when such codes are first exposed to selection. In Figure 8, the mean time to encode ≥20 functions for mechanism #20, 121 passages (Figure 4), is taken as a reference. Figure 8 plots SGC proximity for all eight canyon-bottom mechanisms at that early time, using the same structured list as Figure 4 and Figure 6. Relevant pathway abbreviations again appear above each datum.

**Least selection resolves a fusion effect on accuracy. Figure 8**′s refined distance index resolves canyon pathways. There is a rift in the canyon floor: leftward pathways in Figure 8 are much closer to the SGC than rightward. Consulting topward abbreviations, fusing pathways (#17–20) are much closer to the SGC than non-fusing ones (#21–24). Such evolutions may also employ independent tables or not (tab/notab) and/or may use wobble assignments or not (wob/nowob), but fusing routes remain always closer to the SGC. This resembles prior conclusions [10,13] that identified code fusions as decisive for the rapid appearance of SGC-like codes.

A canyon mechanism worth noting is #24, which relies on non-selective code division alone, nocc div nofus notab nowob. It is significantly slower than #18 and #20 to complete codes (Figure 4) but has a very good overall error (Figure 6) and is deficient only in total SGC proximity (Figure 8). Code division, even acting alone in pathway #24, suffices for moderately rapid code evolution. 

Further, Figure 8 again favors the exclusion of wobble during assignment [3]; as in Figure 4 and Figure 6) and also favors the absence of parallel independent codes (notab) in the two mechanistic environments where it can be compared with a similar path (#17 vs. #19 and also #18 vs. #20).

**Four most competent pathways.** Thus, favored paths to the SGC are defined: via the leftward canyon, nocc div fus. Moreover, the most favored pathway is resolved. That is path #20, nocc div fus notab nowob. 

But, given that choice, tab/notab and wob/nowob options are similar (differing by <<2-fold). At the early times of Figure 8, for example, near-complete codes identical to the SGC using the second-best pathway #18 are 77% the abundance of similar codes via pathway #20. Thus, as a potential SGC pathway, both #18 and #20 must be considered.

**Code division and fusion collaborate, but independently.** It is no surprise that among the most SGC-like codes here, code fusion is frequent. Probabilities were chosen to make fusion effective. However, a new question arises from the introduction of code division. Are division and fusion related or independent features of code evolution? Though no div fus interaction was consciously implemented, human intuition is untrustworthy when so many processes interact.

**Figure 9:**
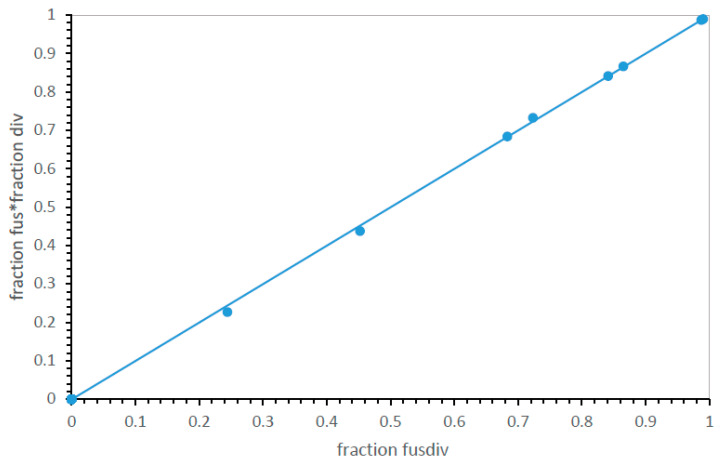
Independence of code fusion and division. Fraction of observed codes using fusion times (indicated with *) fraction of observed codes using division versus fraction observed codes using division and fusion together. Environments are those in Figure 4.

Figure 9 plots the product of the fraction of best codes fusing with the fraction dividing for the 8/32 pathways that use both div and fus and the 24 that do not (plotted at zero). This is compared to the observation: the fraction of best codes employing both fusion and division are counted among results. Figure 9 shows that the product of fraction fus and div and observed conjoined fusdiv in results are virtually identical. Therefore, fusion and newly introduced division aid code evolution, but by acting independently. 

**Figure 10:**
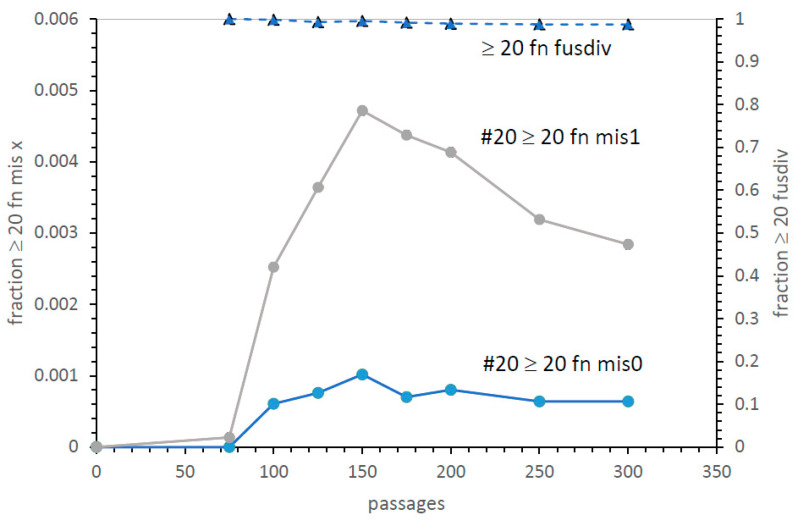
Kinetics of appearance of SGC-like codes. Codes with ≥20 encoded functions and also mis0 or mis1 are plotted versus time in passages. The fraction of ≥20 encoded function codes arising from fusion and division together (dashed line with triangles) is also plotted. Conditions are those for pathway #20: Pmut = 0.00975, Pdec = 0.00975, Pinit = 0.15, Prand = 0.05, Pfus = 0.001, Ptab = 0.0, Pwob = 0.0, Pdivn = 0.25, cc ≥ 1 encoded function.

**A second, more efficient crescendo.** Figure 10 shows early kinetics for the reproducibly superior #20 pathway. In particular, it shows two code species closest to the SGC (≥20 encoded functions with mis0 or mis1). There is a rapid rise after fusion becomes significant, then a prolonged presence of ≥20 assignment codes, zero or one assignment from the SGC. This accurate era lasts hundreds of passages. Thus, there will be many code assignments, decays, captures, fusions, and divisions during this period. Said another way, proficient (Figure 10) codes vary across time, but continuously present novel near-SGC-relatives for selection. 

Moreover, in Figure 10, at the top is the fraction of ≥20 function codes that have assignments from both code fusion and division. Best SGC candidates arise nearly entirely by code division and fusion combined (dashed line, Figure 10). This parallels the succession of highly competent codes from fus alone [10], but this fus div crescendo arises more quickly and yields more frequent SGC-like codes. At the 150-passage peak, there are 1 in 340 live ≥20 function mis0 codes (1 in 1000 total codes, including unsuccessful fusions: Figure 10), or 1 in 74 live ≥20 function mis1 codes (1/210 of total codes: Figure 10). A selection would seem to easily find these relatively frequent SGC-like codes. Therefore, fusion with division is a more probable route to the SGC than fusion alone [13].

## 4. Discussion

**Code division is influential.** In this work, early genetic codes divide to make replicas of themselves. Code divisions are controlled by a probability of division per passage (Pdiv) and independently by a coding threshold (cc) that must be equaled or exceeded to make code division possible. The threshold is inspired by studies showing that 10–13 amino acids must be used in active enzymes and, thus, presumably, to make sophisticated structural proteins needed to support precise division. However, by reducing the threshold to one encoded amino acid, the threshold effect is circumvented—any existing code can then divide. It was thought to study the idea [14] that the early code ensured its evolutionary success by enabling its carrier to divide accurately, founding an evolutionary radiation whose winners had uniquely efficient protein biosynthesis and carried their genetic code to predominance. Such an era has already been persuasively modeled [19].

Though late genetic-code-based radiation is still probable, results here concern an earlier RNA era, before protein AARS—code division profoundly alters early code history. Division speeds SGC evolution itself (Figure 1A,B). The fastest evolution occurs for unselective division when any code can divide (Figure 1A, Figure 3 and Figure 5). Under these conditions, the fastest approach to the SGC yet seen is observed (compare [10]). Moreover, code division reinforces the majority of SGC assignments: when a mixture of SGC and random assignments is supplied, division tends to SGC rather than random assignments (Figure 3). Such preservation increases if division is more likely (increased Pdiv, Figure 1B), as well as with more time to divide (cc = 1: Figure 1A).

**Evolution in parallel.** It was initially thought that code fusion would be advantageous because it allows parallel progress toward the SGC, gathering changes made in different coding compartments instead of waiting for all modifications in a single ancestral line [3]. This can be quantitated (Figure 4 and Figure 8; [10]). Here, in part because of improved fusion, dividing SGC evolution is ≈3-fold accelerated over non-dividing codes fusing with independent genetic codes (Figure 4, Figure 8 and Figure 9). 

**Thirty-two possible pathways to the SGC: rates of evolution.** With the addition of options for a code division threshold (cc/nocc) and division frequency (Pdiv ≥ 0) to previous models, there are 2^5^ = 32 types of pathways for code evolution. In this work, a code evolves entirely without one of these five effects or, in contrast, with a probability known to alter coding outcomes (see the Supplementary Data File). 

**Figure 11:**
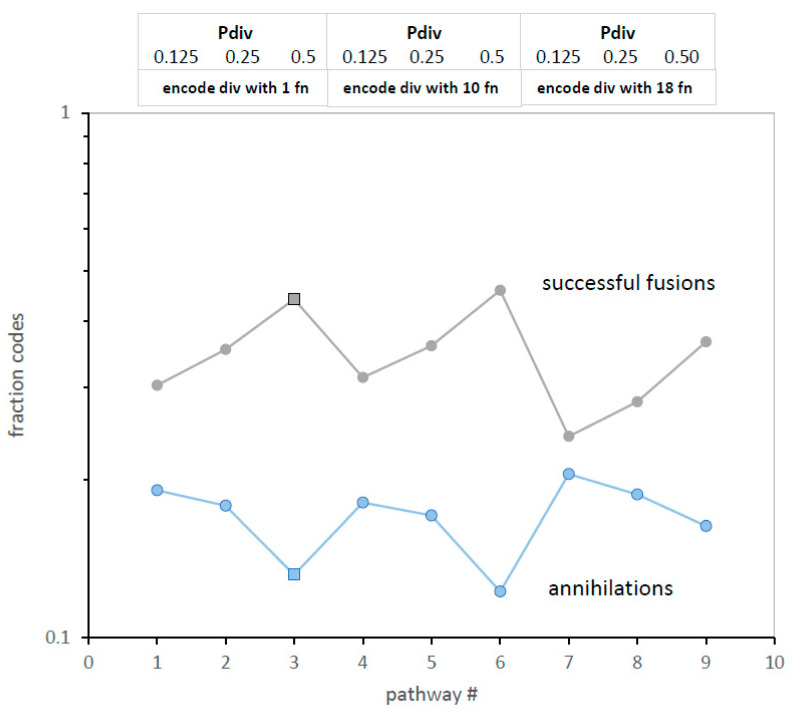
Successful and unsuccessful fusions (annihilations) have complementary behavior. Only code division varies; pathways and conditions are those of Figure 1A, as indicated by abbreviations at plot top. Square points are the same as in Figure 1A,B and Figure 3.

Plotting evolutionary results against a structured list of pathways (Figure 1A, Figure 3, Figure 4, Figure 6 and Figure 11), defined at plot top, multiple different evolutionary pathways can be compared. This is first used for differing division rates and differing thresholds (Figure 1A and Figure 3) and then extended to all 32 pathways (Figure 4 and Figure 6), emphasizing the rate of approach to the complete set of SGC assignments (Figure 4), the adherence of the resulting codes to SGC encoding (Figure 3, Figure 6 and Figure 8) and the role of code division (Figure 11).

The rate of evolution shows a notable canyon of fast evolution (Figure 4) for eight mechanisms (#17–24) that allow code division (div) and impose no threshold for division (nocc). Conspicuously, all eight canyon mechanisms encode ≥20 functions more quickly than any of the other 24 possible pathways.

**Thirty-two possible pathways to the SGC: accuracy of evolution.** There is a general relation between speed and accuracy of code evolution: this is shown in Figure 7, where the times for evolution to ≥20 functions are shown versus accompanying misassignment for 32 pathways. The observation that much of the variance for accuracy can be explained by the rate of evolution is welcome. Figure 7 implies one can find quick evolution accompanied by accurate assignment, so starting from a mixture of initial encodings becomes plausible.

This promise is fulfilled in Figure 4 for rates and Figure 6 for accuracies. These profiles have similar shapes: time to ≥20 functions and misassignments track well for most of the 32 very different mechanisms (Figure 7). Most particularly, a #17–24 canyon with quick evolution and accurate assignments exists in Figure 4 and Figure 6.

**A rift in the canyon floor: the role of fus.** Codes requiring the least selection [14] to become the SGC are likely precursors to the historical code. Thus, further resolution comes from a more precise measure of distance to the SGC, incorporating both speed and accuracy. In Figure 8, such an index is implemented for the eight canyon codes (Figure 4 and Figure 7), using as distance metric the fraction of codes that encode ≥20 functions and are simultaneously accurate: mis0, mis1, or their sum.

Reading the upper legend of Figure 8, there is a large difference in codes that fuse (fus) and those that do not (nofus). Maximally complete codes via fusion (#17–20) are about an order of magnitude more abundant than via non-fusing pathways (#21–24). This parallels previous findings [10,12] that most complete codes come from code fusion. A nocc div fus canyon subset (Figure 8) of pathways implements the doubly capable code evolution implied by the speed-accuracy correlation (Figure 7).

Moreover, the common use of code fusion by #17–20, the four most probable of 32 SGC pathways, supports the necessity of merging primordial codes, initially proposed for other reasons [10,12].

While differences among Figure 8′s complete and accurate codes are not large, pathway #20 (nocc div fus notab nowob) is again superior, implying the least selection to evolve the SGC. 

**Implications of a flat canyon floor.** Differences between tab/notab and wob/nowob codes are dramatically curtailed within the nocc div canyon, where these variations have their smallest observed effects (Figure 4 and Figure 7). Such small effects are of evolutionary importance in two ways. 

The first relates to wobble: how can one rationalize the universal adoption of wobble coding when it is everywhere unfavorable (see **Wobble is always inhibitory** above)? One response is that wobble is likely delayed [12], but another is that there exist pathways (#17–20, Figure 4, Figure 7 and Figure 9) where wobble has a minimal negative effect. Wobble introduced late in pathway #18 or 20 would not be selected against.

**Comparing the best pathways**. Small canyon-floor differences between tab/notab pathways are also evolutionarily significant. Routes #18 and 20 host codes that reach the SGC most quickly (Figure 4) while also preserving high accuracy (Figure 6). When speed and accuracy are required together (Figure 8), #18 and 20 are again best. What does this multiple superiority mean? 

Figure 8 shows that #18 and #20 environments differ only for independent codes—it is somewhat better to avoid them. This is puzzling because more independent codes provide a broader sample of the coding environment and are generally expected to find the SGC sooner [13]. Moreover, multiple codes can fuse, quickly forming more complete codes by summing compatible assignments [10,12]. Figure 8, therefore, suggests that something subtle makes path #20 (nocc div fus notab nowob) best, and in particular, superior to #18 (nocc div fus tab nowob).

**Multiple codes are more advantageous if they resemble each other.** The subtlety is in the nature of “other” codes. When independent codes fuse, they assemble complete, accurate code tables significantly more rapidly [10]. However, code division creates a new kind of fusion partner. Figure 11 illustrates this, using a pathway containing both independent and division fusion partners (Figure 1A). As code division increases in Figure 11, the fraction of codes with successful fusion among environmental codes increases. Even more relevantly, unsuccessful fusions (annihilations via conflicting assignment) decrease, and by the same proportion as fusion increase. Figure 11′s two plots mirror each other. Especially apt fusion partners from code division replace fusion to independently arising codes to make up the approximately constant number of assignments required for complete code construction (Figure 1A). At all levels of code division, the quickest (Figure 1A) and most accurate SGC-like evolution (Figure 3) is associated with the greatest successful (Figure 11, square) and least unsuccessful (Figure 11) code fusion.

With time, dividing, highly related code numbers increase, so variants of a dividing code will be made and tested more rapidly. This resembles the ‘crescendo’ of competent codes created by fusion with increasing numbers of unrelated codes [10]. In this work, novel partner codes originate by division and subsequent evolution, but the result is similar: an era when highly complete, highly accurate codes proliferate. SGC selection can survey a second kind of prolonged fusion-division crescendo (Figure 11), during which many different but related SGC-like codes are exposed to selection. 

**Pathway #20 simplifies SGC evolution**. Thus, the “disadvantage” of fusion between independent codes is only that a better path exists: a dividing code population harvests evolutionary change by fusing evolved ancestral codes and varied descendants. Most especially, this effect makes evolution from a unique origin (pathway #20) somewhat more efficient than fusing with independent codes (pathway #18). Simpler primordial code emergence by the least SGC selection from a single ancestor is plausible.

**Fusion yields hybrid routes to the SGC. Figure 1**′s varying division rates move code evolution along an axis joining pathways #18 and #20. Code division increases, independent code fusion decreases (toward #20), or the reverse (toward #18) with a small change in result (Figure 8). Hybrid routes with similar SGC access suggest novel possibilities. Specifically, pathways #18 and 20 approach the SGC by fusing early coding tables from differing origins. Therefore, these pathways suggest that partial codes from other origins could be fused. 

The SGC can have an even earlier history [20], but the early code usually becomes structured in one of four ways. ‘Frozen accidents’: Crick [21] supposed that a code could be frozen, perhaps after being shaped by earlier molecular interactions. In any case, a growing code would ultimately become difficult to change because changes would perturb all previous gene products [22]. ‘Coevolution’: reference [23] emphasizes that it is undeniable that code progress could have been shaped by metabolic evolution, more complicated amino acids encoded only after progressive biosynthesis reaches them. This is a highly developed theory [24,25,26,27] often called coevolution of the genetic code. ‘Error minimization’: a code or partial code might be shaped by selection to minimize the effects of coding errors or mutations [28,29]. Strikingly, error minimization can arise without selection against error [30]. ‘Stereochemistry’: coding assignments might reflect the chemical interaction of amino acids and ribonucleotides. Selected RNA binding sites for amino acids contain an excess of anticodon and codon triplets. Each triplet is an essential sequence for amino acid binding, as shown by sequence conservation and mutagenesis data [31,32,33]. Genomic sequencing [34,35] suggests that related interactions can still be seen throughout modern mRNAs.

Notably, all mechanisms could yield code fragments that fuse. Even more to this point, divergent mechanisms plausibly utilize varied sets of triplets. Codes from disparate origins could have fewer overlapping, conflicting assignments. As shown here for independent codes versus dividing codes (Figure 8 and Figure 11), efficient evolution results when code fusion is less failure-prone. To summarize, the SGC can originate from a single source via fusions and divisions or from multiple sources via fusions.

**Biology as anthology**. Inspired by the calculated advantages of code fusion, it was suggested that life can be defined by facile gathering of separate advantages into one line of descent [12]. From this work, we add that code evolution has effortlessly combined advantages (Figure 4, Figure 6 and Figure 7) and also will automatically refine gathered advantages (Figure 11). Division and fusion are elementary cellular activities; thus, code refinement has a simple, almost inevitable, rationale. Such powerful cellular effects were probably not used solely to create the SGC.

## Data Availability

See the Supplementary Data File, Table S1: RFW_supp_data_a.xlsx. The author will entertain requests for further data.

## Supplementary Materials

The Table of supporting information can be downloaded at: https://www.mdpi.com/article/10.3390/life13102069/s1, Table S1: RFW_supp_data_a.xlsx.
